# A deep learning-based radiomics model for predicting lymph node status from lung adenocarcinoma

**DOI:** 10.1186/s12880-024-01300-w

**Published:** 2024-05-24

**Authors:** Hui Xie, Chaoling Song, Lei Jian, Yeang Guo, Mei Li, Jiang Luo, Qing Li, Tao Tan

**Affiliations:** 1 Department of Radiation Oncology, Affiliated Hospital (Clinical College) of Xiangnan University, Chenzhou, Hunan province 423000 People’s Republic of China; 2 Faculty of Applied Sciences, Macao Polytechnic University, Macao, 999078 People’s Republic of China; 3 School of Medical Imaging, Laboratory Science and Rehabilitation, Xiangnan University, Chenzhou, Hunan province 423000 People’s Republic of China; 4 Department of Radiology and Nuclear Medicine, Radboud University Medical Centre, Nijmegen, Netherlands

**Keywords:** Radiomics, Deep learning, Machine learning, Lymph node metastasis, Lung adenocarcinoma, Model

## Abstract

**Objectives:**

At present, there are many limitations in the evaluation of lymph node metastasis of lung adenocarcinoma. Currently, there is a demand for a safe and accurate method to predict lymph node metastasis of lung cancer. In this study, radiomics was used to accurately predict the lymph node status of lung adenocarcinoma patients based on contrast-enhanced CT.

**Methods:**

A total of 503 cases that fulfilled the analysis requirements were gathered from two distinct hospitals. Among these, 287 patients exhibited lymph node metastasis (LNM +) while 216 patients were confirmed to be without lymph node metastasis (LNM-). Using both traditional and deep learning methods, 22,318 features were extracted from the segmented images of each patient's enhanced CT. Then, the spearman test and the least absolute shrinkage and selection operator were used to effectively reduce the dimension of the feature data, enabling us to focus on the most pertinent features and enhance the overall analysis. Finally, the classification model of lung adenocarcinoma lymph node metastasis was constructed by machine learning algorithm. The Accuracy, AUC, Specificity, Precision, Recall and F1 were used to evaluate the efficiency of the model.

**Results:**

By incorporating a comprehensively selected set of features, the extreme gradient boosting method (XGBoost) effectively distinguished the status of lymph nodes in patients with lung adenocarcinoma. The Accuracy, AUC, Specificity, Precision, Recall and F1 of the prediction model performance on the external test set were 0.765, 0.845, 0.705, 0.784, 0.811 and 0.797, respectively. Moreover, the decision curve analysis, calibration curve and confusion matrix of the model on the external test set all indicated the stability and accuracy of the model.

**Conclusions:**

Leveraging enhanced CT images, our study introduces a noninvasive classification prediction model based on the extreme gradient boosting method. This approach exhibits remarkable precision in identifying the lymph node status of lung adenocarcinoma patients, offering a safe and accurate alternative to invasive procedures. By providing clinicians with a reliable tool for diagnosing and assessing disease progression, our method holds the potential to significantly improve patient outcomes and enhance the overall quality of clinical practice.

**Supplementary Information:**

The online version contains supplementary material available at 10.1186/s12880-024-01300-w.

## Introduction

Lung cancer is one of the leading causes of cancer-related death worldwide [1]. According to the American Cancer Society (https://www.cancer.org), 350 people die of lung cancer every day in the United States in 2022 [2]. Furthermore, lung cancer is also the malignant neoplasm with the highest incidence and mortality in China [3]. Lung adenocarcinoma comprises about 40% of all lung cancer cases [2]. Thanks to advanced computed tomography techniques of the chest and low-dose screening with computed tomography (CT), many lung cancer patients are detected at an early stage. The early diagnosis of lung cancer is of great significance to improve the treatment level and prognosis of patients. The National comprehensive cancer network (NCCN) guidelines state that surgery should be the first option for patients with early lung cancer [4]. However, lung tumors often involve mediastinal lymph nodes. Lymph node metastasis rates have been reported in 15% to 20% of patients with early-stage non-small cell lung cancer(NSCLC) whose lung tumors are 2 cm or less in diameter [5]. Lymphatic metastasis is the most common metastatic pathway in lung cancer. Clinically, the presence of lymph node metastasis (LNM) is a crucial factor in determining the TNM staging of lung cancer patients. The TNM staging system, which stands for Tumor, Node, and Metastasis, is a widely used method for classifying the severity and extent of lung cancer. Within this system, the status of the lymph nodes plays a pivotal role in assessing the overall clinical stage of the disease, thereby guiding treatment decisions and predicting patient outcomes [6]. Preoperative assessment of the presence of LNM in lung cancer patients can provide valuable information for determining the need for adjuvant therapy and surgery, thus helping clinicians make the right decision.

At present, there are many methods to evaluate LNM in lung cancer patients, including CT, Positron Emission Tomography-CT(PET-CT), ultrasound-guided biopsy, thoracoscopy, etc. [7]. Although biopsy and thoracoscopy can better evaluate lymph node staging, both are invasive. The radiologist determines the LNM status by the size of the lymph nodes on the CT, which obviously has great limitations [8]. PET-CT is a relatively accurate imaging technique with a high specificity for LNM in preoperative lung cancer patients. However, the misdiagnosis and false negative rates of LNM diagnosed by PET-CT are high [9]. In addition, PET-CT scans are often too expensive for most patients, particularly in less economically developed countries, which further restricts their widespread clinical application.

As a well-established field, radiomics can play an important role in the preoperative evaluation of LNM in lung cancer patients [10–12]. Radiomics extracts quantifiable features from medical images, such as intensity, texture, and shape descriptors. These features capture the heterogeneity within the tumor, which is often associated with its biological behavior and response to treatment. By analyzing these features, radiomics can assist in diagnosing the presence of malignancy, evaluating the prognosis of patients, and predicting the likely outcome of different treatment options. This non-invasive approach offers clinicians a powerful tool for personalized medicine, enabling more precise and targeted care for lung cancer patients. Yang et al. [10] collected 159 lung adenocarcinoma patients for radiomics analysis and established a prediction model of LNM with an AUC of 0.86. Zheng et al. [11] collected radiological characteristics and clinical parameters of 217 patients with stage I-IIIB NSCLC to predict LNM status, and the test set AUC was 0.71. Huang et al. [12] recruited 155 patients with NSCLC and established a PET-CT radiomics model to predict LNM, with an AUC of 0.847. But we found this kind of studies are based on small data set, and the number of positive cases of low, large sample distribution bias. There are also a large number of studies that lack external test sets when constructing models.

This study aims to achieve seamless integration of radiomics with deep learning(DL), leveraging the efficient feature extraction capabilities of DL to facilitate the development of accurate predictive models, in order to achieve new breakthroughs in the medical field. At the same time, we aim to explore the potentially optimal model through attempting various machine learning modeling methods, and to construct a lung adenocarcinoma lymph node metastasis prediction model by mining radiomics features from CT images. This model has been validated using external data and proven to have clinical auxiliary diagnostic value.

## Patients and methods

**Fig. 1:**
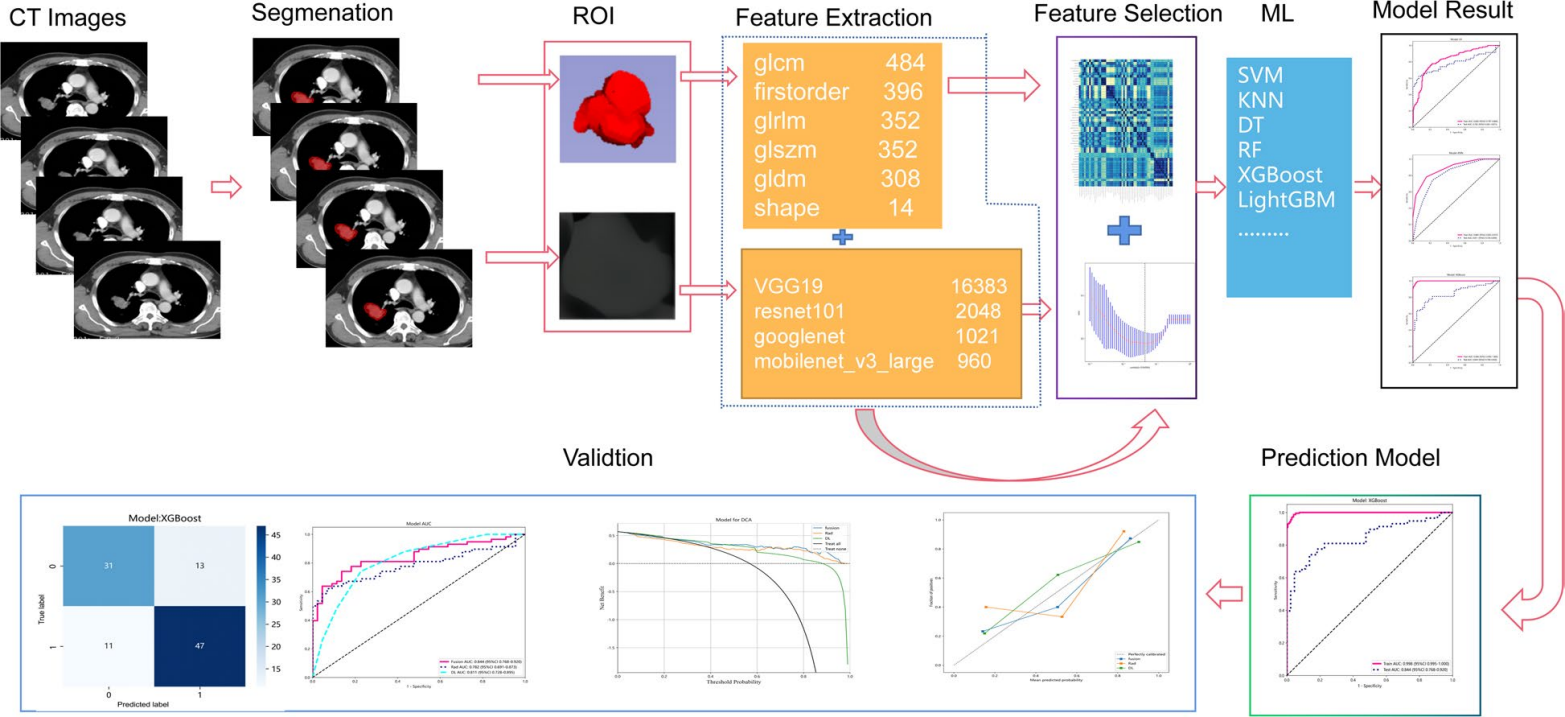
Map of technical route. GLCM: Gray-Level Co-occurrence Matrix; GLRLM: Gray-Level Run-Length Matrix; GLSZM: Gray-Level Size Zone Matrix; GLDM: Gray-Level Dependence Matrix

This study has obtained formal approval from the Institutional Review Board (IRB) of two participating institutions. These institutions are the Medical Research Ethics Committee of Xiangnan University Affiliated Hospital (Clinical College) (Approval Number: AF/SC-07–4/05.0) and the Medical Research Ethics Committee of the People's Hospital of HeBi (Approval Number: 22–350-18018). Both institutions have waived the need for informed consent for this study. This study was conducted following ethical guidelines of World Medical Association (WMA) Declaration of Helsinki. The technology roadmap for the whole study is shown in Fig. 1. The details are as follows.

### Data collection

**Fig. 2:**
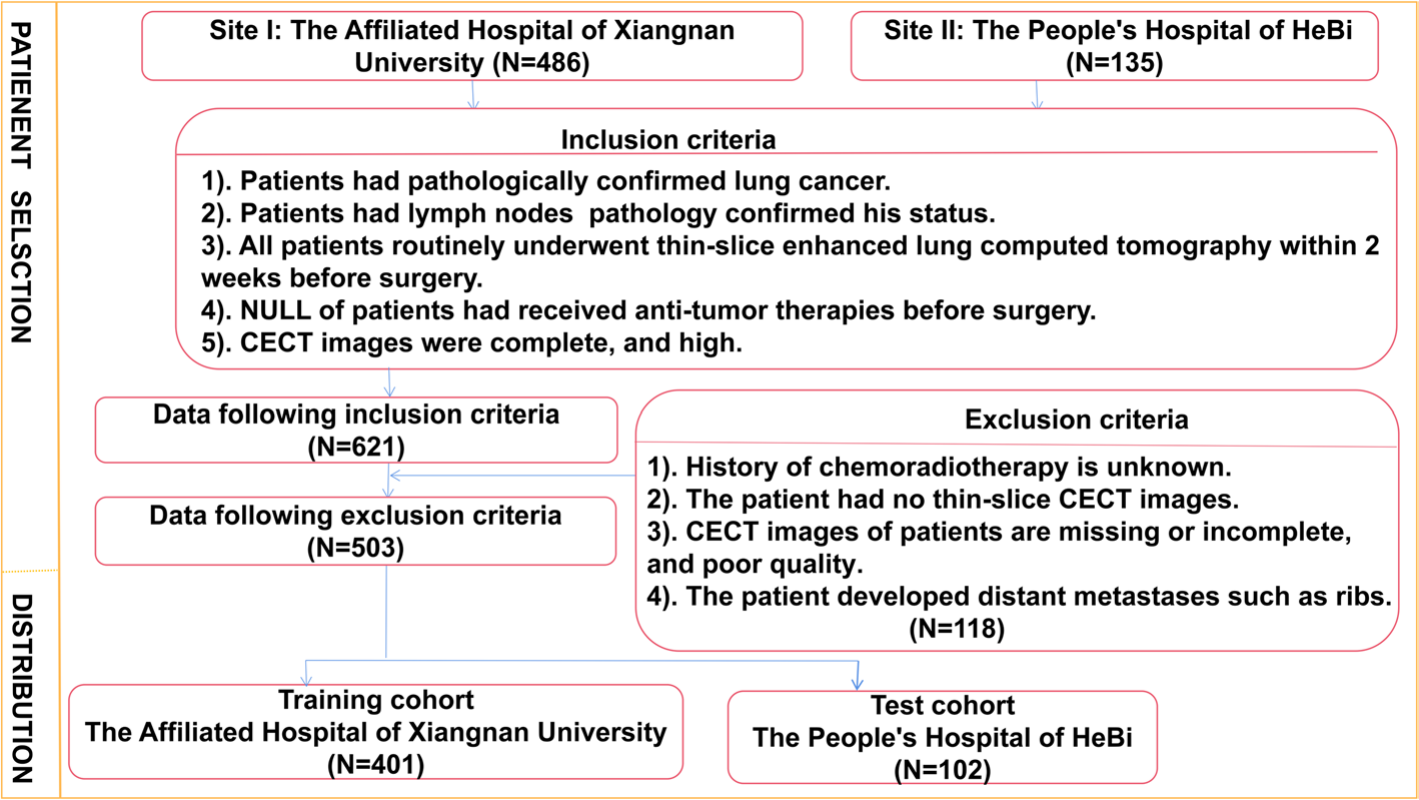
Inclusion and exclusion criteria for patient selection for the training and test cohorts. In total, 503 of 621 patients were enrolled in this study according to the selection criteria. CECT: Contrast-enhanced Computed Tomography

A total of 621 patients with lung adenocarcinoma confirmed by pathological results who underwent contrast-enhanced CT (CECT) thin-layer scanning in the Affiliated Hospital (Clinical College) of Xiangnan University (*N* = 486) and the People's Hospital of HeBi (*N* = 135) from January, 2017 to May, 2022 were retrospectively collected. From this, 503 patients were selected. The flow chart of the screening datasets is presented in Fig. 2. All enrolled patients were divided into two groups: 287 patients with lymph node metastasis (LNM +) and 216 patients without lymph node metastasis (LNM-). The Affiliated Hospital of Xiangnan University cohort was selected as the training set and the People's Hospital of HeBi cohort was selected as the test set. Inclusion criteria were as follows: (1). Patients had pathologically confirmed lung adenocarcinoma. (2). Dissection of lymph nodes and pathology showed lymph node status. (3). All patients in our study underwent routine thin-slice CECT of the lungs, with a 5mm slice thickness acquisition that was later reconstructed to 1–1.5mm slices, within a two-week window before their surgical procedure. (4). NULL of patients had received chemotherapy, radiotherapy and other related anti-tumor therapies before surgery. (5). CECT images were complete, and 3D-slicer software could be used to delineate lung cancer lesions smoothly and accurately. The exclusion criteria are as follows: (1). History of chemoradiotherapy is unknown. (2). The patient had no thin-slice CECT images. (3). CT images of patients are missing or incomplete, and the quality of CT images is inadequate. (4). The patient developed distant metastases such as ribs. In this study, all interpretations of CT images were conducted by experienced radiologists specializing in diagnostic radiology, who possess at least 15 years of professional experience.

### CT scan protocols

Thin-slice CECT scan was performed on all patients. The screening scanner are the second generation Siemens SOMATOM HD FlashCT scanner (The Affiliated Hospital of Xiangnan University, Somatom Definition, Siemens Medical Solutions, Forchheim, Germany) and the Lighspeed-16 row scanner of GE Company (The People's Hospital of HeBi, GE Healthcare, Waukesha, WI, USA). All patients were examined in the supine position, and the scan range was from the thorax entrance to the posterior costal angle. The single breath was continuously screened after end-inspiratory. Scan parameters and settings were as follows: pitch (1.0mm), tube voltage (120 KVp), and tube current (80–300 mA). All CT image display settings were as follows: Lung window (level of − 600HU and width of 1200HU), mediastinal window (level of 40 HU and width of 350 HU). For the CECT scans, 1.5–2 ml/kg of non-ionic contrast media iohexol (100 ml, Jiangsu Hengrui Medicine Co., Ltd., Nanjing, China) was injected through the elbow vein at a rate of 3.0–3.5ml/s. The scan phase imaging was venous (delay, 90 s). All CT images were exported in Digital Imaging and Communications in Medicine (DICOM) format for radiomics feature extraction.

### Data acquisition

Segmentation of the lung tumor region of interest (ROI): Lung tumors were manually delineated on vein-phase CECT by two radiologists with at least 15 years of experience in a blinded manner using the "segmenteditor" in 3D-Slicer (version 4.6, https://www.slicer.org) [13]. Then, a radiologist with the title of the associate chief physician or above reviewed and revised the tumor contours on CECT images one by one as the final gold standard for delineation. The purpose of this was to reduce the variability of manual delineation. If a controversial delineation was encountered, the three people discussed to decide how to delineate the tumor contour. After the manual segmentation, all images were resampled voxel size to 3 × 3 × 3 mm^3^ for the next step.

The "Pyradiomics" package [14] was used to extract the traditional radiomics features of the lung 3D ROI in python environment. The “Pyradiomics” package incorporates various filters, including exponential, LBP, logarithm, square, and wavelet, to extract diverse quantitative features from medical images. The exponential filter emphasizes specific image regions, LBP captures local texture patterns, logarithm stabilizes data variance, square normalizes data, and wavelet analyzes information across multiple scales. These filters jointly contribute to accurate and efficient feature extraction, enhancing clinical decision-making and research outcomes [14]. The extracted the traditional radiomics features comprehensively characterize the lung ROI. The Gray-Level Co-occurrence Matrix (GLCM) captures spatial relationships between pixel values, effectively quantifying various texture properties. First-Order Features detail the distribution of pixel values, including mean, median, and standard deviation (SD), providing a comprehensive overview of intensity characteristics. The Gray-Level Run-Length Matrix (GLRLM) quantifies the length of consecutive pixels, revealing lung texture uniformity and complexity. The Gray-Level Size Zone Matrix (GLSZM) analyzes the size of connected regions, offering insights into the spatial distribution of lung tissues. The Gray-Level Dependence Matrix (GLDM) explores the dependency between gray levels and their spatial arrangement, capturing intricate patterns and structures. Finally, Shape Features describe the geometric properties of the lung ROI, such as compactness and elongation, providing a holistic representation of its morphological features.

The maximum cross-sectional slices were obtained from the 3D ROI of the lung, and then the averge pool layer of the four DL models [15, 16] (VGG19 [17], resnet101 [18], googlenet [19] and mobilenet_v3_large [20]) were used to extract DL radiomics features. Four machine learning models underwent preliminary training using the 'ImageNet' dataset [21], followed by further training with CT images to enhance their specialization for medical image analysis.

### Model construction

The Affiliated Hospital of Xiangnan University cohort (*N* = 401) was selected as the training set and the People's Hospital of HeBi cohort (*N* = 102) was selected as the test set. The training set was used to build a prediction model and the test set was used to test this model. Data normalization and filtration were performed prior to statistical analysis. Then, the least absolute shrinkage and selection operator (LASSO) was adopted. L1 regularization shrinks coefficients of less important features to zero by adding the absolute value of magnitude of coefficients as a penalty term to the loss function. Finally, we used support vector machine (SVM), K-nearest neighbor (KNN), randomforest (RF), extreme gradient boosting (XGBoost), light gradient boosting machine (LightGBM), NaiveBayes (NB), adaptive boosting (AdaBoost), gradient boosting (GBDT), logistic regression (LR) and multilayer perceptron (MLP) 10 machine learning algorithms were used to establish classification prediction models by hyperparameter method. The Rad_score of the model can be calculated as follows:

1 $$Rad\_Score = \sum\limits_{i} {{\text{feature}}_{{\text{i}}} \times {\text{Coefficient}}} + b$$

Cofficient is obtained by an iterative of LASSO algorithm, and i represents the feature.

### Statistical analysis

Python(version: 3.7, http://www.python.org [22]) was used for statistical analysis. Continuous variables were expressed as mean ± standard deviation ($$\begin{gathered} \overline{x} \pm s \hfill \\ \hfill \\ \end{gathered}$$), and categorical variables were expressed as counts. Differences between groups were analyzed using *t*-tests and Chi-squared tests. Consistency assessment of radiomics features: The CT images of 50 randomly selected patients were segmented and radiomics features were extracted by the same radiologist twice, with an interval of more than one month. The intra-group (ROI segmentation of 50 patients by two radiologists, respectively) and inter-group (ROI segmentation of 50 patients by two radiologists, respectively) were used. Intraclass Correlation Coefficient (ICC) method was used to evaluate the repeated consistency of features. Features are extracted consistently when ICC is higher than 0.75 [23, 24]. Omics features are standardized with Z-score, and the formula is as follows:

2 $${\mathrm X}_\_\mathrm{norm}\;=\frac{\left(\mathrm X\;-\;\mathrm{mean}\right)}{\mathrm{std}}$$

Spearman correlation method was used to preliminary screen the features. Travel through the LASSO algorithm L1 penalty function has been screened again, filtering features built into the model in the end. The classification prediction model was established by using 10 machine learning algorithms (SVM, KNN, RF, XGBoost, LightGBM, NB, AdaBoost, GBDT, LR and MLP). The diagnostic performance of the model was evaluated by Area under the curve (AUC), Accuracy, Specificity, Precision, Reall and F1 score.

3 $${\text{Accuracy}} = \frac{{{\text{(TP}} + {\text{TN)}}}}{{{\text{(TP}} + FN + FP + TN)}}$$

4 $$Specificity = \frac{TN}{{TN + FP}}$$

5 $${\text{Precision}} = \frac{{{\text{TP}}}}{{{\text{(TP}} + {\text{FP)}}}}$$

6 $${\text{Recall}} = \frac{{{\text{TP}}}}{{{\text{(TP}} + {\text{FN)}}}}$$

7 $${\text{F1}} = \frac{{{\text{2TP}}}}{{{\text{(2TP}} + {\text{FP}} + {\text{FN)}}}}$$

*TP *True Positive, *FN *False Negative, *FP *False Positive, *TN *True Negative

Decision curve analysis (DCA) was used to test whether the predictive model was clinically significant. In the training set, five-fold cross validation was used to determine the stability of the model, and then all the data from the test set were used to build a complete model to develop the final prediction model. The Hosmer–Lemeshow test was used to generate calibration curves to test whether the predicted results was consistent with the actual results. For each model, the AUC values were tested by Delong tests (*p* < 0.01 was considered statistically significant).

In the above analysis, there is no special explanation that *P* < 0.05 indicates statistical difference.

## Results

### Repeatability assessment of feature extraction

After manual segmentation by 3D-slice software. In the python environment, 1906 traditional radiomics features were extracted from each patient, and the ICC values of intra-group and inter-group were 0.786–0.962 and 0.783–0.833, respectively, which were greater than 0.75, indicating good consistency.

### Traditional radiomics feature selection and model construction

**Fig. 3:**
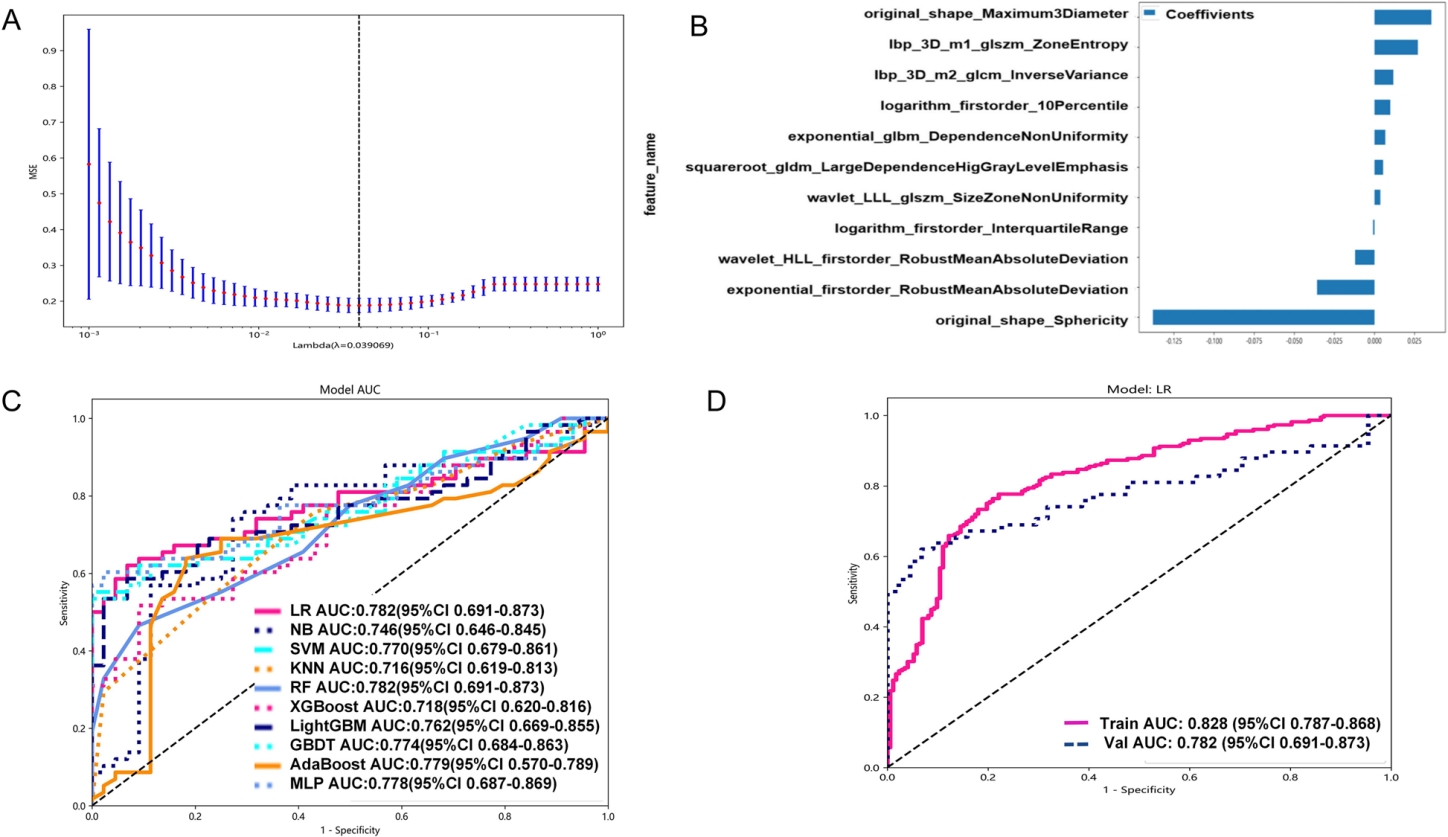
Rad-modle construction. **A** Lasso screening of Rad-model predictor variables; **B** Bar plot showing the regression coefficients for the hub feature of Rad-modle; **C** ROC plot for 10 ML models in test set of Rad-modle. **D** ROC plot for Rad-modle in training and test set

There were 401 cases in the training set, including 172 cases of LNM(-) and 229 cases of LNM( +). In the test set of 102 cases, including 44 cases of LNM(-) and 58 cases of LNM( +). Supplementary Table 1 provides further clinical information on the enrolled patients, including age, gender, and details regarding the primary tumor site. Additionally, CT characteristics such as lobulation and Burr sign are also presented in this supplementary Table 1 for a more thorough understanding of the patients' condition. The training set included 1906 traditional radiomics features, and 364 features were obtained after spearman test (threshold was set to 0.9). LASSO was used to further reduce the dimension, when λ = 0.039 (Fig. 3A) the 11 best traditional radiomics features (Fig. 3B) were obtained. Using ten kinds of machine learning algorithms, constructing classification prediction model respectively. The performance evaluation of the ten classification prediction models across both the training and test sets is comprehensively presented in Table 1. Upon careful analysis of the ROC curve depicted in Fig. 3C, it becomes evident that the LR model exhibits superior prediction capabilities among the traditional radiomics-based prediction models. As highlighted in Fig. 3D and summarized in Table 1, LR stands out as the most effective in terms of overall performance. Unfortunately, we observed instances of overfitting in the XGBoost and RF models. This overfitting may have hindered their ability to generalize effectively to unseen data. Furthermore, several other models demonstrated suboptimal performance across various metrics, including Specificity, Precision, Recall, and F1. Therefore, based on the traditional radiomics features, LR algorithm was used to construct a prediction classification model (Rad-modle).

**Table 1:** The results of 10 traditional radiomics model analysis

Model		Accuracy	AUC	Specificity	Precision	Recall	F1
Train	LR	0.763	0.828	0.674	0.772	0.830	0.800
	NB	0.738	0.803	0.750	0.795	0.729	0.761
	SVM	0.788	0.855	0.732	0.805	0.830	0.817
	KNN	0.786	0.871	0.715	0.797	0.838	0.817
	RF	0.982	0.998	0.977	0.983	0.987	0.985
	XGBoost	0.990	0.999	0.994	0.996	0.987	0.991
	LightGBM	0.843	0.919	0.791	0.849	0.882	0.865
	AdaBoost	0.788	0.868	0.826	0.853	0.760	0.804
	GBDT	0.818	0.900	0.785	0.839	0.843	0.841
	MLP	0.778	0.836	0.773	0.821	0.782	0.801
Test	LR	0.696	0.782	0.614	0.721	0.759	0.739
	NB	0.725	0.746	0.636	0.742	0.793	0.767
	SVM	0.667	0.770	0.568	0.694	0.741	0.717
	KNN	0.676	0.716	0.568	0.698	0.759	0.727
	RF	0.657	0.729	0.500	0.672	0.776	0.720
	XGBoost	0.647	0.718	0.545	0.677	0.724	0.700
	LightGBM	0.696	0.762	0.682	0.745	0.707	0.726
	AdaBoost	0.706	0.679	0.750	0.780	0.672	0.722
	GBDT	0.657	0.774	0.636	0.709	0.672	0.690
	MLP	0.696	0.778	0.705	0.755	0.690	0.721

*SVM* Support vector machine, *KNN* K-nearest neighbor, *RF* Randomforest, *XGBoost* Extreme gradient boosting, *LightGBM* Light gradient boosting machine, *NB* NaiveBayes, *AdaBoost* Adaptive boosting, *GBDT* Gradient boosting, *LR* Logistic regression, *MLP* Multilayer perceptron

### Deep learning radiomics feature selection and model construction

**Fig. 4:**
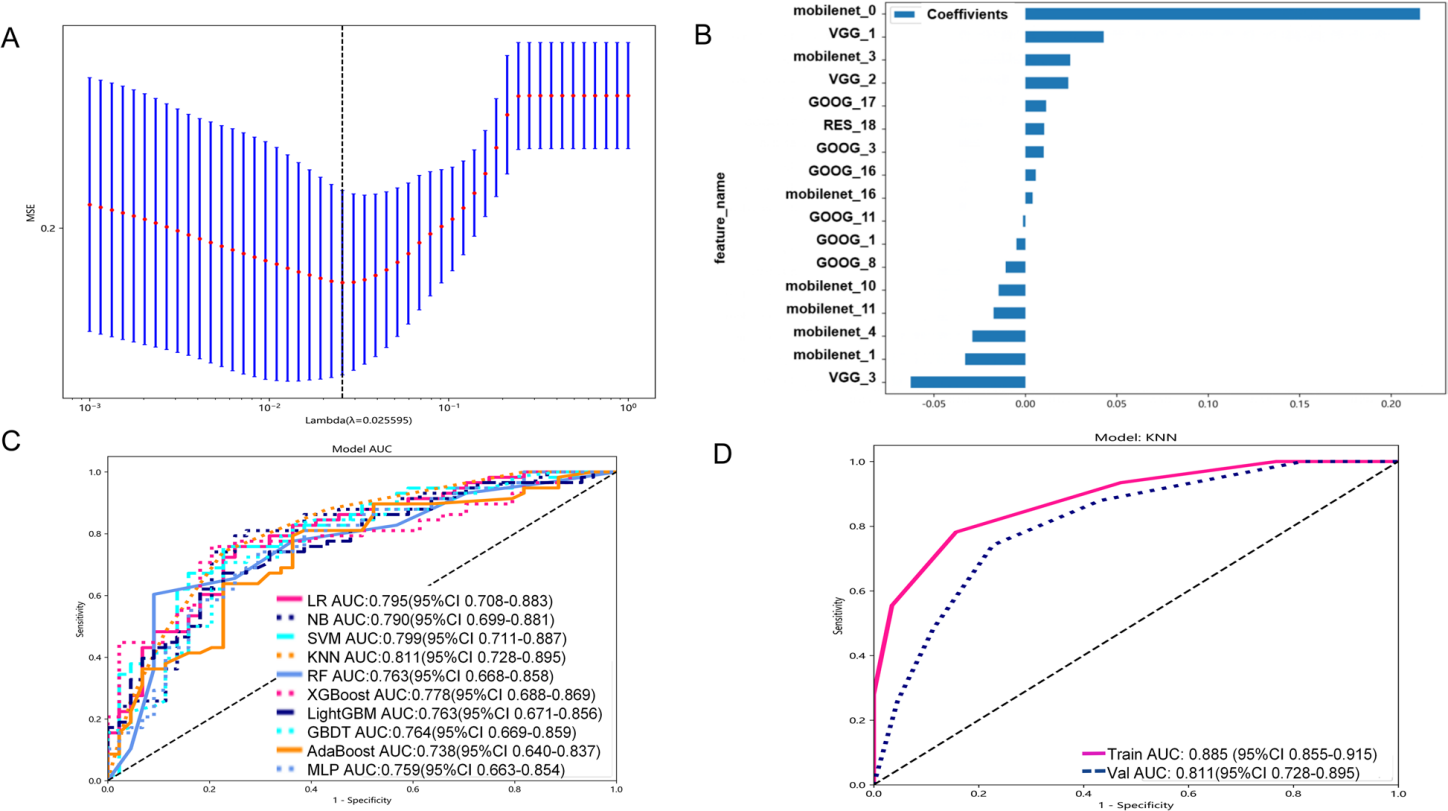
DL-modle construction. **A** Lasso screening of DL-model predictor variables; **B** Bar plot showing the regression coefficients for the hub feature of DL-model; **C** ROC plot for 10 ML models in test set of DL-model. **D** ROC plot for DL-modle in training and test set

The maximum cross section of ROI of each patient was fed to four DL models (vgg19, resnet101, googlenet and mobilenet_v3_large) for DL radiomics feature extraction. Vgg19 extracted 16,383 features; resnet101 extracted 2,048 features; googlenet extracted 1,021 features; mobilenet_v3_large extracted 960 features. A total of 20,412 DL radiomics features were obtained. By spearman correlation (threshold was set to 0.9) and LASSO (λ = 0.013, Fig. 4A), finally got 17 DL radiomics features, used for model building (Fig. 4B). Table 2 shows the performance of the 10 machine learning classifiers in the training and test sets. The ROC curves of each classification prediction model in the test set are shown in Fig. 4C.

**Table 2:** The results of 10 deep learning radiomics model analysis

Model		Accuracy	AUC	Specificity	Precision	Recall	F1
Train	LR	0.758	0.869	0.727	0.792	0.782	0.787
	NB	0.778	0.838	0.802	0.837	0.760	0.797
	SVM	0.850	0.936	0.826	0.869	0.8696	0.869
	KNN	0.808	0.885	0.843	0.869	0.782	0.823
	RF	0.985	0.999	0.971	0.979	0.996	0.987
	XGBoost	0.988	0.995	0.994	0.996	0.983	0.989
	LightGBM	0.860	0.949	0.791	0.853	0.913	0.882
	AdaBoost	0.803	0.881	0.756	0.821	0.838	0.829
	GBDT	0.838	0.931	0.756	0.831	0.890	0.864
	MLP	0.805	0.902	0.703	0.798	0.882	0.838
Test	LR	0.735	0.795	0.705	0.772	0.759	0.765
	NB	0.765	0.790	0.750	0.804	0.776	0.789
	SVM	0.735	0.799	0.614	0.738	0.828	0.780
	KNN	0.755	0.811	0.773	0.811	0.741	0.775
	RF	0.657	0.763	0.432	0.658	0.828	0.733
	XGBoost	0.735	0.778	0.682	0.763	0.776	0.769
	LightGBM	0.676	0.763	0.568	0.698	0.759	0.727
	AdaBoost	0.716	0.738	0.591	0.72	0.810	0.764
	GBDT	0.725	0.764	0.636	0.742	0.793	0.76
	MLP	0.716	0.759	0.614	0.730	0.793	0.760

*SVM* Support vector machine, *KNN* K-nearest neighbor, *RF* Randomforest, *XGBoost* Extreme gradient boosting, *LightGBM* Light gradient boosting machine, *NB* NaiveBayes, *AdaBoost* Adaptive boosting, *GBDT* Gradient boosting, *LR* Logistic regression, *MLP* Multilayer perceptron

Similar to the traditional radiomics model (Table 1), the RF and XGBoost models exhibited signs of overfitting. The NB, AdaBoost, GBDT, and MLP models did not perform as well as the KNN model. In the test set, the KNN model exhibited comparable performance to both LR and SVM. However, when it comes to metrics such as Accuracy, AUC, Specificity, Precision, and Recall, KNN emerged as the top performer. While KNN slightly trailed behind LR and SVM in terms of the F1, its overall excellence in the majority of evaluation metrics makes it the preferred choice as the final model. Through comprehensive analysis, KNN model based on DL radiomics features performs best. Therefore, based on these DL radiomics features, KNN algorithm was used to construct a prediction classification model (Fig. 4D, DL-modle).

### Comprehensive predictive model construction

**Fig. 5:**
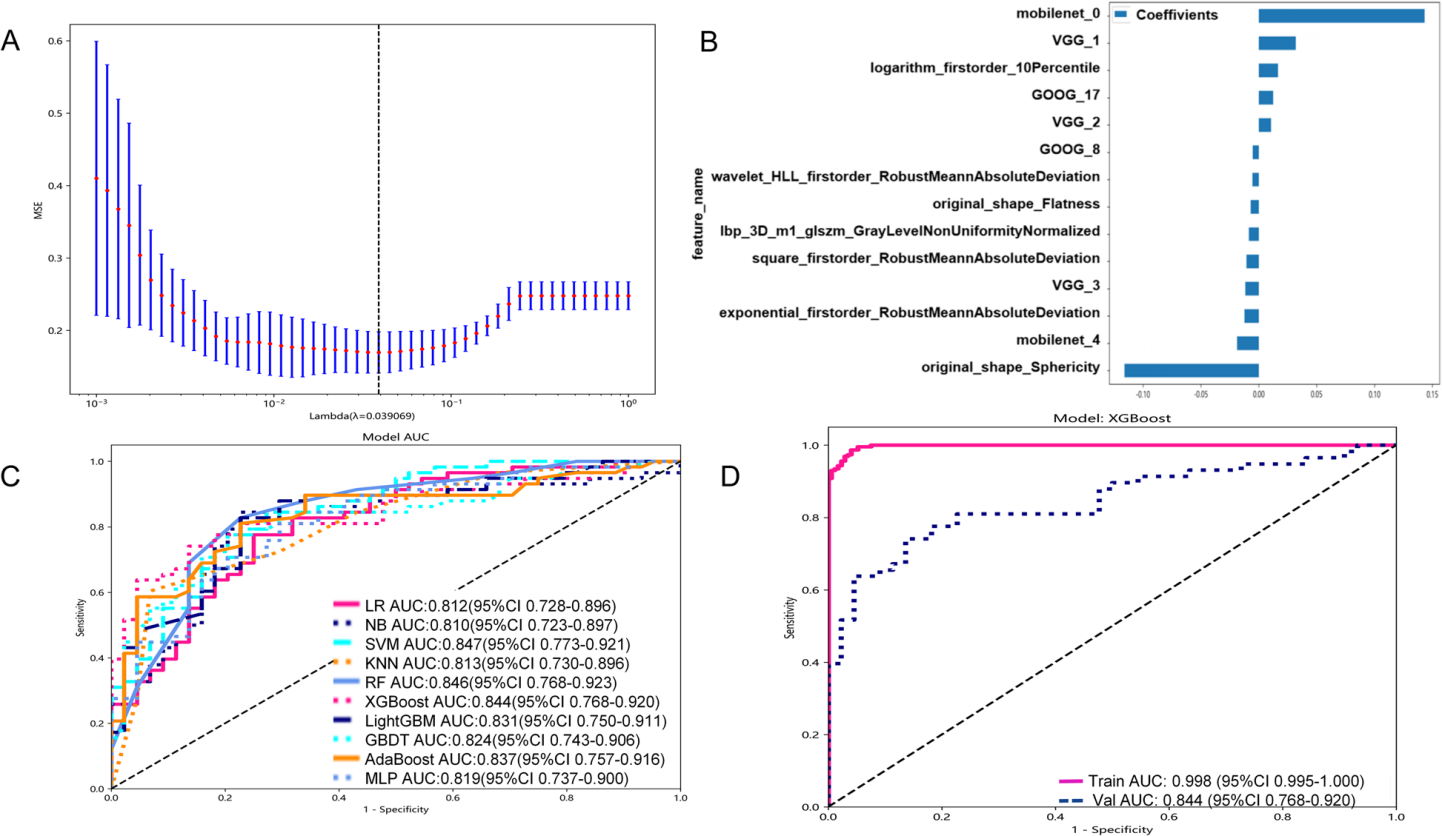
Fusion-modle construction. **A** Lasso screening of Fusion-model predictor variables; **B** Bar plot showing the regression coefficients for the hub feature of Fusion-model; **C** ROC plot for 10 ML models in test set of DL-model. **D** ROC plot for Fusion-modle in training and test set

The extracted traditional radiomics and DL radiomics features were integrated. By spearman correlation (threshold was set to 0.9) and LASSO (λ = 0.039, Fig. 5A), ends up with 14 hub features (Fig. 5B). There were 7 traditional radiomics features: Exponential_firstorder_RobustMeanAbsoluteDeviation, lbp_3D_m1_glszm_GrayLevelNonUniformityNormalized, logarithm_firstorder_10Percentile, original_shape_Flatness, original_shape_Sphericity, Square_firstorder_RobustMeanAbsoluteDeviation and wavelet_HLL_firstorder_RobustMeanAbsoluteDeviation. And, there were 7 DL radiomics features: VGG_1, VGG_2, VGG_3, GOOG_8, GOOG_17, mobilenet_0 and mobilenet_4. Among the 7 DL radiomics features, VGG_1, VGG_2 and VGG_3 were derived from the DL model of VGG19. GOOG_8, GOOG_17 are derived from DL model of googlenet, mobilenet_0 and mobilenet_4 are derived from DL model of mobilenet_v3_large. However, features extracted from DL model of restnet101 were not significantly correlated with LNM of lung adenocarcinoma. These 7 traditional radiomic features are classified into first-order features, gray-level size zone matrix (GLSZM) and shape features. Using these 14 hub features, the classification prediction model were constructed by 10 machine learning algorithms. From Table 3, as well as in the ROC curve (Fig. 5C), we can find XGBoost performance is superior to other models of the model. Therefore, based on the traditional and DL radiomics features, XGBoost algorithm was used to construct a prediction classification model (Fusion-modle, Fig. 5D).

**Table 3:** The results of 10 prediction model analysis, basis on the traditional and deep learning radiomics

Model		Accuracy	AUC	Specificity	Precision	Recall	F1
Train	LR	0.803	0.898	0.738	0.813	0.852	0.832
	NB	0.763	0.8638	0.651	0.764	0.847	0.803
	SVM	0.850	0.940	0.814	0.863	0.878	0.870
	KNN	0.803	0.895	0.802	0.844	0.804	0.823
	RF	0.995	0.997	0.994	0.996	0.996	0.996
	XGBoost	0.968	0.998	0.959	0.969	0.974	0.972
	LightGBM	0.880	0.953	0.814	0.869	0.930	0.899
	AdaBoost	0.808	0.907	0.814	0.852	0.803	0.827
	GBDT	0.855	0.934	0.843	0.880	0.865	0.872
	MLP	0.805	0.902	0.704	0.798	0.882	0.838
Test	LR	0.745	0.812	0.682	0.767	0.793	0.780
	NB	0.765	0.810	0.659	0.766	0.845	0.803
	SVM	0.794	0.847	0.727	0.803	0.845	0.824
	KNN	0.716	0.813	0.705	0.764	0.724	0.743
	RF	0.784	0.846	0.659	0.773	0.879	0.823
	XGBoost	0.764	0.844	0.705	0.783	0.810	0.797
	LightGBM	0.784	0.831	0.727	0.800	0.828	0.814
	AdaBoost	0.775	0.837	0.773	0.818	0.775	0.796
	GBDT	0.775	0.824	0.705	0.787	0.828	0.807
	MLP	0.716	0.759	0.614	0.730	0.793	0.760

*SVM* Support vector machine, *KNN* K-nearest neighbor, *RF* Randomforest, *XGBoost* Extreme gradient boosting, *LightGBM* Light gradient boosting machine, *NB* NaiveBayes, *AdaBoost* Adaptive boosting, *GBDT* Gradient boosting, *LR* Logistic regression, *MLP* Multilayer perceptron

### Prediction model determination

**Fig. 6:**
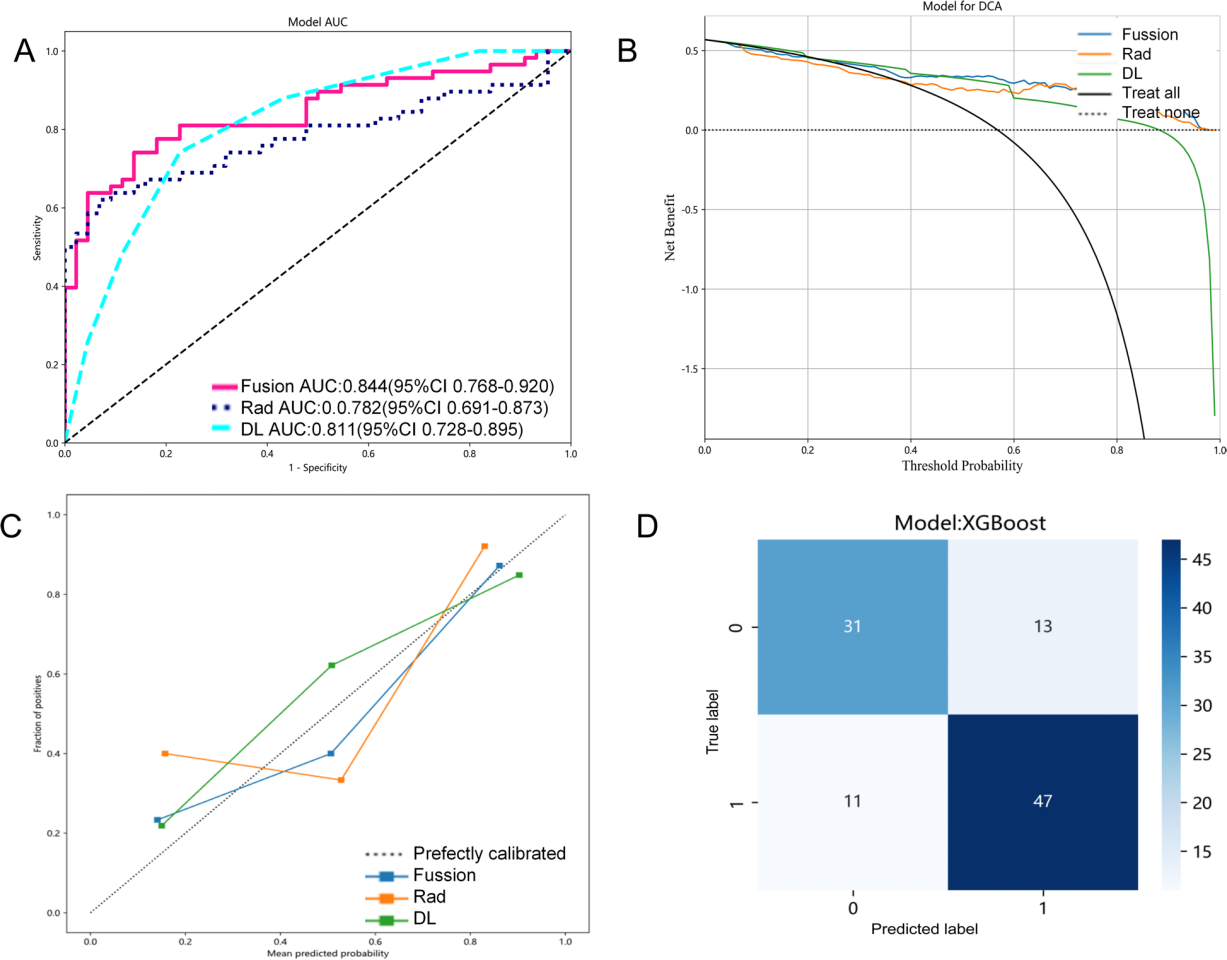
Fusion-model validation in the validation cohort. **A** ROC curve of the validation set; **B** DCA for the model in the test cohort; **C** Calibration curves of the model in the test cohort; (**D**) Confusion matrix for the test set of Fusion-model. In (**D**), 1 represents LNM( +) and 0 represents LNM(-)

Despite partial overlap in the confidence intervals depicted in Fig. 6A, upon comprehensive evaluation of various metrics such as prediction accuracy, AUC value, and model stability (Tables 1, 2, and 3), coupled with consideration of practical application needs, we have identified the XGBoost method, leveraging both traditional and DL radiomics features, as exhibiting superior performance across multiple dimensions. Notably, the XGBoost method demonstrates significant advantages over other methods in terms of Accuracy, AUC, Specificity, Precision, Recall, and F1. Consequently, within the context of this study, we can confidently assert that the XGBoost method possesses relatively superior predictive performance. The DCA curve (Fig. 6B) of the three models showed that the fusion model also had the greatest clinical benefit. Therefore, this study identified this model as the final predictive classification model. The calibration curve (Fig. 6C) of the model in the test set showed that the model has strong applicability and high prediction accuracy. As shown in Fig. 6D, the normalized confusion matrix further showed the model’s classification accuracy on the test set.

In addition, the Rad score of this model to predict the LNM of lung adenocarcinoma patients can be calculated as:

Rad_score = 0.5716628665724426.

-0.012359 × exponential_firstorder_RobustMeanAbsoluteDeviation.

-0.008611 × lbp_3D_m1_glszm_GrayLevelNonUniformityNormalized.

 + 0.016666 × logarithm_firstorder_10Percentile.

-0.006898 × original_shape_Flatness.

-0.116110 × original_shape_Sphericity.

-0.010594 × square_firstorder_RobustMeanAbsoluteDeviation.

-0.005452 × wavelet_HLL_firstorder_RobustMeanAbsoluteDeviation.

 + 0.031910 × VGG_1.

 + 0.010605 × VGG_2.

-0.011560 × VGG_3.

-0.005295 × GOOG_8.

 + 0.012453 × GOOG_17.

 + 0.143270 × mobilenet_0.

-0.018727 × mobilenet_4.

Although the results of the DeLong's test reveal no statistically significant difference between the ROC curves of the Fusion-model, Rad-model, and DL-model (Fusion-model *VS* Rad-model [*p*-value = 0.116], Fusion-model *VS* DL-model [*p*-value = 0.499], DL-model *VS* Rad-model [*p*-value = 0.609]), we maintain that the Fusion-model emerges as the superior model in this study due to its seamless integration of diverse techniques or methodologies. This integration potentially enhances generalization capabilities, stability, and reduces specific error types, albeit these benefits did not achieve statistical significance in the ROC curve analysis. Nonetheless, the Fusion-model remains a frontrunner worthy of further investigation and application within our research framework. Its superiority is further underscored by its performance across various evaluation metrics, including Accuracy, Specificity, Precision, Recall, F1, DCA, and calibration curves.

## Discussion

LNM is a crucial factor for clinicians to determine the clinical staging of lung cancer, formulate treatment plans, and predict prognosis [6]. Although current medical imaging examination can detect LNM to some extent, an assessment solely on morphological changes is insufficient to provide accurate histopathological information. There is an urgent need for a non-invasive and effective method to evaluate the LNM status of patients. This study compares the traditional, DL, and DL-traditional radiomics models in predicting LNM based on preoperative CECT of lung adenocarcinoma. In cases with larger datasets, DL models have outperformed hand-crafted feature extraction [25]. However, access to large data in the field of medicine is relatively difficult and may be affected by disease prevalence rates, data acquisition, and other clinical factors [26]. For smaller datasets, studies have shown that feature engineering may be more suitable for machine learning strategies [27], and radiomics has advantages in medical imaging analysis. Currently, there are relatively few studies that directly compare the performance of radiomics and DL models [14, 28, 29]. In this study, we predicted the risk of LNM in lung adenocarcinoma patients. We not only verified that the DL-based prediction model (with accuracy, AUC, specificity, precision, recall and F1 in the test set are as follows: 0.755, 0.811, 0.773, 0.811, 0.741and 0.775) was superior to the traditional radiomics model (with accuracy, AUC, specificity, precision, recall and F1 in the test set are as follows: 0.696, 0.782, 0.614, 0.721, 0.759, 0.739), but also the Fusion model (Accuracy, AUC, Specificity, Precision, Recall and F1 in the test set are as follows:0.765, 0.844, 0.705, 0.783, 0.810 and 0.797) obtained by integrating DL and traditional radiomics had better prediction results and improved model interpretability to a certain extent. The DCA curve of external validation intuitively shows that the Fusion model has higher clinical benefit than the other two models. Moreover, the calibration curve verified by external verification proves that the Fusion model is in good agreement with the actual value.

Among the hub features for constructing Fusion model, 7 are traditional radiomics features. In radiomics RobustMeanAbsoluteDeviation is all intensity and gray level between or equal to the average between the 10th and the 90th percentile of the distance between the average, and negatively correlated with metastasis [30]. Our study also confirmed this theory. And in our study RobustMeanAbsoluteDeviation has carried on the wavelet, exponential and square transformation makes the futertes of the multiple correction, and stronger stability [31]. Shen et al. [28]. reported that the RobustMeanAbsoluteDeviation feature played a key role in distinguishing histological subtypes of NSCLC. In addition, Sphericity is also introduced into the modeling. Our study also suggests that Sphericity is negatively correlated with LNM of lung adenocarcinoma. In other words, the more regular and spherical the primary tumor shape, the less likely it is to cause LNM. This is consistent with the studies of others, where smaller Sphericity is more likely to induce LNM in breast cancer and esophageal cancer [29]. In clinical practice, an important feature for radiologists to read CT to judge lung tumors is the burr-like structure [31], which also represents the irregularity of the tumor. Burr-like structure of NSCLC is more aggressive and has poor prognosis [32]. In Supplementary Table 1, it can be observed that there is also a significant difference in Burr sign between the two groups, NLM( +) and NLM(-). It can be inferred that the smaller of Sphericity, the higher malignant degree of lung adenocarcinoma and the greater possibility of LNM. The 10Percentile is the set of intensity voxels in the region of interest, which represents less than 10% of the observations [33]. This research shows that, by the 10Percentile can predict the nature of the lesion, and positive correlation. This means that in the CT pulmonary primary lesion is on the gray scale difference exist in whether LNM. However, the gray difference in the specific area of the lesion needs further research. The study by Folhoffer et al. [34] pointed out that the 10Percentile and the 90Percentile are very useful for the classification of high and low grade fibrosis in the liver. Shen's study also showed that 10Percentile can be used to classify the subtypes of lung cancer [28]. GLSZM is the starting point of the Thibault matrix, which can effectively describe the texture uniformity, non-periodicity or similar texture [35]. It has been proved that the gray level quantization has an important effect on the texture classification performance [36]. The gray level non-uniformity is a radiological texture feature that indicates heterogeneity [37]. It is particularly important that many radiomics features are unstable among different reconstruction algorithms, and GLNU is one of the most reproducible radiomics features with good stability [38]. Recent studies have shown that the value of GLNU increases if the lesion is heterogeneous [35]. Heterogeneity is an important feature of malignant tumors [39], which is closely related to the malignant biological behavior, and can reflect the changes of related growth factors and the microenvironment of tumor growth [40]. The higher the malignant degree of tumors, the higher the heterogeneity [41]. In the study of Yang X et al. [10], GLNU was incorporated into the prediction of LNM status of lung adenocarcinoma, and the AUC in the training and test set were 0.854 and 0.803, respectively. In a previous study, GLNU was also identified as the most important radiomics features in hypertrophic cardiomyopathy [42].

However, in this study, we not only used traditional radiomics features, but also used DL methods. Four DL models (VGG19, resnet10, Googlenet and mobilenet_v3_large) were used to extract DL radiomics features. The integration of DL radiomics features into feature engineering has greatly increased the dimensionality of the data studied (from the original 1,907 dimensions to the later 22,319). Finally, 14 hub features (7 traditional radiomics features, 7 DL radiomics features) were entered into the construction of the model. This method can make the prediction results more reliable. Our study dataset is relatively large, with 503 patients from CECT images routinely acquired in clinical settings, which greatly improves the authenticity of the results. Unlike most current studies, our study used an external data test set. A total of 102 patients from the People's Hospital of HeBi were used as the test set to verify the model. The prediction AUC of the model reached 0.844 in the test set, which had strong robustness. While the Fusion model demonstrates significant superiority in various metrics such as Accuracy, Specificity, Precision, Recall, and F1 score, surpassing both the traditional radiomics model and the DL model, it is important to acknowledge that it does have some limitations. Notably, in the Delong test, the Fusion model did not achieve the desired level of performance. This could be attributed to various factors, including the complexity of the dataset, the specific nature of the test, or potential areas for improvement in the model's architecture or training process. Despite this shortcoming, the Fusion model's overall excellence in other metrics remains compelling and suggests that it holds great potential for further development and optimization.

Nonetheless, it is imperative to recognize that our research possesses inherent limitations that necessitate additional scrutiny and consideration. Firstly, given the strong association between the LNM of lung adenocarcinoma and genetic factors [43], we recognize the need to incorporate genomics data in future studies. The absence of such data in our current investigation limits our understanding of the underlying genetic mechanisms involved in LNM. Secondly, the incomplete follow-up data precluded us from conducting thorough investigations into patient outcomes, which is crucial for assessing the long-term impact of our findings. To address these limitations, we plan to explore various avenues in future research. Firstly, our primary objective is to refine the architecture and hyperparameters of the machine learning model in order to bolster its predictive capabilities. This might involve exploring various neural network structures and meticulously adjusting the learning rate to achieve optimal performance. Secondly, we intend to investigate the generalizability of our model by applying it to different cancer datasets, thereby extending its potential applications to other malignancies. Finally, we are interested in exploring the integration of our model with other clinical and genetic data to develop a more comprehensive and personalized approach to cancer diagnosis and treatment. By addressing these limitations and exploring these directions, we hope to contribute to the advancement of precision medicine in cancer treatment.

## Conclusion

Leveraging enhanced CT images, our study introduces a noninvasive classification prediction model based on the extreme gradient boosting method. This approach exhibits remarkable precision in identifying the lymph node status of lung adenocarcinoma patients, offering a safe and accurate alternative to invasive procedures. By providing clinicians with a reliable tool for diagnosing and assessing disease progression, our method holds the potential to significantly improve patient outcomes and enhance the overall quality of clinical practice.

### Supplementary Information

Supplementary Material 1.

## Data Availability

The datasets used and/or analysed during the current study available from the corresponding author on reasonable request.
