# Supplementary material for: A deep learning-based radiomics model for predicting lymph node status from lung adenocarcinoma
Source: BMC Med Imaging. 2024 May 24;24:121. doi: 10.1186/s12880-024-01300-w (PMC11127329; doi:10.1186/s12880-024-01300-w)
Supplement: Supplementary file 1 — Supplementary Material 1. [file 12880_2024_1300_MOESM1_ESM.docx]

Supplementary Table 1

Patients and preoperative clinical feature

| Clinical features | Training group (n=401) | | P |  | Test group (n=102) | | P |
| --- | --- | --- | --- | --- | --- | --- | --- |
|  | LNM(-)  (n=172) | LNM(+)  (n=229) |  |  | LNM(-)  (n=44) | LNM(+)  (n=58) |  |
| Age (Mean± SD) | 61.03±8.13 | 59.26±11.34 | 0.361 |  | 61.56±8.94 | 57.62±13.26 | 0.572 |
| Range | 38-87 | 36-78 |  |  | 43-78 | 41-82 |  |
| Gender |  |  | 0.753 |  |  |  | 0.312 |
| Male | 93 | 111 |  |  | 15 | 25 |  |
| Female | 79 | 118 |  |  | 29 | 33 |  |
| Primary lung cancer site |  |  | 0.632 |  |  |  | 0.263 |
| Upper lobe, right | 71 | 93 |  |  | 18 | 23 |  |
| Middle lobe, right | 24 | 32 |  |  | 5 | 6 |  |
| Lower lobe, right | 12 | 22 |  |  | 3 | 3 |  |
| Upper lobe, left | 42 | 51 |  |  | 12 | 18 |  |
| Lower lobe, left | 23 | 31 |  |  | 6 | 8 |  |
| Number of the primary tumors |  |  |  |  |  |  |  |
| Single | 128 | 139 | 0.003 |  | 32 | 39 | 0.049 |
| Multiple | 44 | 90 |  |  | 12 | 19 |  |
| Smoke |  |  | 0.586 |  |  |  | 0.851 |
| Yes | 69 | 94 |  |  | 18 | 32 |  |
| No | 93 | 135 |  |  | 26 | 26 |  |
| Lobulation |  |  | 0.026 |  |  |  | 0.038 |
| Yes | 121 | 166 |  |  | 35 | 43 |  |
| No | 51 | 63 |  |  | 9 | 15 |  |
| Burr |  |  | 0.019 |  |  |  | 0.004 |
| Yes | 116 | 173 |  |  | 33 | 46 |  |
| No | 56 | 56 |  |  | 11 | 12 |  |
